# Microneedle Enhanced Delivery of Cosmeceutically Relevant Peptides in Human Skin

**DOI:** 10.1371/journal.pone.0101956

**Published:** 2014-07-17

**Authors:** Yousuf H. Mohammed, Miko Yamada, Lynlee L. Lin, Jeffrey E. Grice, Michael S. Roberts, Anthony P. Raphael, Heather A. E. Benson, Tarl W. Prow

**Affiliations:** 1 Dermatology Research Centre, The University of Queensland, School of Medicine, Translational Research Institute, Brisbane, Queensland, Australia; 2 School of Pharmacy, CHIRI-Biosciences, Curtin University, Perth, Western Australia, Australia; 3 Therapeutics Research Centre, The University of Queensland, School of Medicine, Princess Alexandra Hospital, Brisbane, Queensland, Australia; Faculdade de Medicina da Universidade de Lisboa, Portugal

## Abstract

Peptides and proteins play an important role in skin health and well-being. They are also found to contribute to skin aging and melanogenesis. Microneedles have been shown to substantially enhance skin penetration and may offer an effective means of peptide delivery enhancement. The aim of this investigation was to assess the influence of microneedles on the skin penetration of peptides using fluorescence imaging to determine skin distribution. In particular the effect of peptide chain length (3, 4, 5 amino acid chain length) on passive and MN facilitated skin penetration was investigated. Confocal laser scanning microscopy was used to image fluorescence intensity and the area of penetration of fluorescently tagged peptides. Penetration studies were conducted on excised full thickness human skin in Franz type diffusion cells for 1 and 24 hours. A 2 to 22 fold signal improvement in microneedle enhanced delivery of melanostatin, rigin and pal-KTTKS was observed. To our knowledge this is the first description of microneedle enhanced skin permeation studies on these peptides.

## Introduction

Peptides have important applications in modulating of skin cell proliferation, cell migration, inflammation, angiogenesis, melanogenesis, and protein synthesis and regulation [1]. With variations in amino acid sequence, number of amino acids and derivatives, the future of peptide based cosmeceuticals is bright [2]. There are three main categories of cosmeceutical peptides: signal peptides, carrier peptides, and neurotransmitter-affecting peptides. The active ingredient must be delivered to the target in stable form and be able to have the desired biological effect in vivo.

The most prevalent and widely used single peptide is lysine-threonine-threonine-lysine-serine (KTTKS) found on type I procollagen. Melanostatin is a novel pseudo-tripeptide with a molecular formula of C_19_H_25_N_5_O_5_ and is structurally related to feldamycin. Melanostatin inhibited melanin formation in Streptomyces bikiniensis NRRLB-1049 and B16 melanoma cells. Rigin is a four amino acid peptide that is can reduce inflammation. Specifically, rigin has been shown to down regulate IL-6 [3], [4].

The transdermal peptide delivery has attracted interest due to the many biological advantages associated with including avoidance of the first-pass metabolism and sustained therapeutic action. However the stratum corneum barrier has been the greatest challenge for transdermal peptide delivery researchers. Thus, many approaches have been attempted to overcome the skin barrier and enhance the transdermal delivery of peptides for local and systemic effects. The major approaches for enhancing transdermal delivery are physical enhancers, vesicles particulate systems and chemical enhancers. The use of microneedles has been used to overcome the stratum corneum barrier. Microneedles are minimally invasive devices that can drug into or through the skin barrier. Microneedles are generally shorter than 1 mm in length and can breach the stratum corneum barrier. One challenge researchers working in this field face is skin elasticity [5]. Hence microneedle length, manner of insertion and application speed govern the shape and size of the pore formed [6].

The amount of time the pores stay open has been an area of constant debate. Bal *et al.* claim a fast closure of the pores by using a confocal laser scanning microscopy (CLSM) to visualise the amount of a fluorescent dye present in the pores. Visualisation using CLSM is one way to obtain information on morphological parameters of the pores and to monitor the behaviour of a pore and the dye over time. Bal *et al.* reported a quick closure of the pores as there was a strong decrease of the dye present at the skin surface after 10–15 min [6]. Banks *et al.* utilized transepidermal water loss (TEWL) measurements after microneedle treatment and microscopic visualization to determine pore lifetime. They also measured skin permeability of NTXOL (naltrexone analogue 6-β-naltrexol) over time to determine the pore lifetime. In addition, a staining technique was developed to microscopically visualize microneedle created pores in treated guinea pig skin. Banks *et al.* concluded that microneedle-assisted transdermal delivery appears viable for at least 48 h after microneedle-application [7]. In a subsequent study they showed that the addition of a COX inhibitor, like diclofenac, can keep the microneedle pores open for up to a week [8]. Transport of drug molecules after microneedle application is hypothesized to take place by simple diffusion [9].

The aim of the present study was to evaluate the distribution of fluorescently-tagged peptides, melanostatin, rigin and palmitoyl-pentapeptide (Pal-KTTKS) after microneedle based delivery using CLSM and to determine distribution of the peptides within the skin strata. In particular the effect of peptide chain length (3, 4, 5 amino acid chain length) on passive and microneedle facilitated skin penetration was investigated.

## Materials and Methods

### 2.1 Skin preparation for permeation studies

Human skin was obtained from abdominoplasty patients. All patients signed an informed consent. This study is specifically approved by the Princess Alexandra Hospital Research Committee approval No. 097/090, administered by the University of Queensland Human Ethics Committee (Australia). The subcutaneous fat was removed by dissection and the full thickness skin then stored at −20°C until required. Skin from different donors was used to demonstrate reproducibility of the study. Before commencing the study the skin was thawed to room temperature and carefully dabbed with clean tissue paper to remove excess moisture. The skin was then cut using a circular die to fit Franz cells.

### 2.2 Microneedles and Microneedle applicator

The microneedles used in this study were cut from a 50 µm thick 304 stainless steel sheet using a LaserPro S290 laser etcher. The microneedle arrays were cut onto a single plate with 700 µm length×250 µm width. Each plate consisted of 3 microneedles separated by a 5 mm distance. These plates were then assembled in banks of 2 with a 3 mm spacing in between. The microneedles were cut in batches with a strict quality control cut-off of 5% standard deviation. A typical batch when observed for quality assurance had an average height of 703.1±16.1 µm and an average width of 257.8±9.4 µm.

The applicator developed for this study was designed to impact the skin surface at 1.5 m/s. After firmly placing the applicator against the skin, the trigger was released to apply the six microneedles.

### 2.3 Microneedle enhanced peptide delivery in human skin

Microfabrication techniques ensured that the microneedles were long enough to cross the permeability barrier (700 µm) but not so long that they are painful [10]. The peptides used in this study were selected as cosmetic and therapeutic peptides with increasing chain length and increasing molecular weight. Fluorescein conjugated peptides melanostatin (PLG; MW 803.92 Da; XLOGP calculated log P−1.3), rigin (GQPR; MW 959.04 Da; XLOGP calculated log P−7.7) and Pal-KTTKS (MW 1191.06 Da, log P 3.5) were first dissolved in appropriate solvents. All peptides were purchased from Genscript (Hong Kong). Eight groups were tested in triplicate. Negative controls were vehicle only and vehicles post microneedle application to account for any autofluorescence. Sodium fluorescein, at equal molar levels to that present in the peptide, was used as a positive control in both passive treatment group where microneedles were not applied, and in active treatment group where microneedles were applied (data not shown). The last two treatments included the peptides with and without microneedles applied at a 500 µg/mL concentration. The duration of the study was 1 hour for all treatments. For the peptides a separate 24 hour time point from the same skin donor was also conducted. The replicate experiments on each peptide were conducted using skin from three donors.

### 2.4 Skin sample confocal microscopy analysis

After completion of the treatment time the skin samples were cut to a smaller size. Before the samples were imaged with CLSM, a dermoscopic image was first taken to identify the exact location of the microneedle fissures. The sample was then mounted with a cover slip. The VivaScope 2500 Multilaser (Caliber I.D., Rochester NY USA) was used for this study as imaging an area large enough to cover the microneedle holes could be done in one tiled image. The excitation wavelength was 445 nm with the laser power (6.8 mW) held constant for all experiments. The images were generated as cubes of tiled images of 8×8 individual images over 20 layers in depth. The cube was generated over 100 µm with images at a step of 5 µm. Similarly cubes were also generated from the same area (depth and location) in reflectance mode. The reflectance image in conjunction with the z-axis profile of the intensity depth was used to determine the top layer of the skin sample. The top 20 µm were designated as the stratum corneum (SC), the following 30 µm were designated as the viable epidermis (VE) and the images from below this (50 µm + deeper) were designated as the dermis (DER) [11]. Mosiacs at 30 and 50 µm were subjected to image analysis and reported as viable epidermis and dermis, respectively.

### 2.5 Image analysis

Image signal intensity analysis was carried out using Image J software NIH (USA). The raw mosaic images were resized to 10% of the original. The threshold value (54) was derived from pilot experiments with where sodium fluorescein was applied to untreated and microneedle treated skin. The images were opened in ImageJ and a threshold of applied. A circular selection (diameter 750 µm and area 4.42×10^5^ µm^2^) was centered on the microneedle site. Similarly, the same diameter circular selection was applied to the non-microneedle treated skin groups. The positive area and integrated density values were generated using the measurements function in ImageJ for all images.

### 2.6 Statistical analysis

The skin permeation data consisted of normalised penetration area and integrated density measurements of active, passive, negative control and positive control taken at 1 and 24 hours within the skin layers, viable epidermis and dermis. Statistical analysis was conducted using GraphPad Prism 5.03 software (GraphPad Software Inc. USA). Mann Whitney test was used to generate *p* values when comparing two data sets. Significant differences were defined as *p*<0.05. Values for integrated density and positive area are shown as mean ± standard deviation.

## Results

### 3.1. Penetration of microneedle in excised human skin

**Figure 1:**
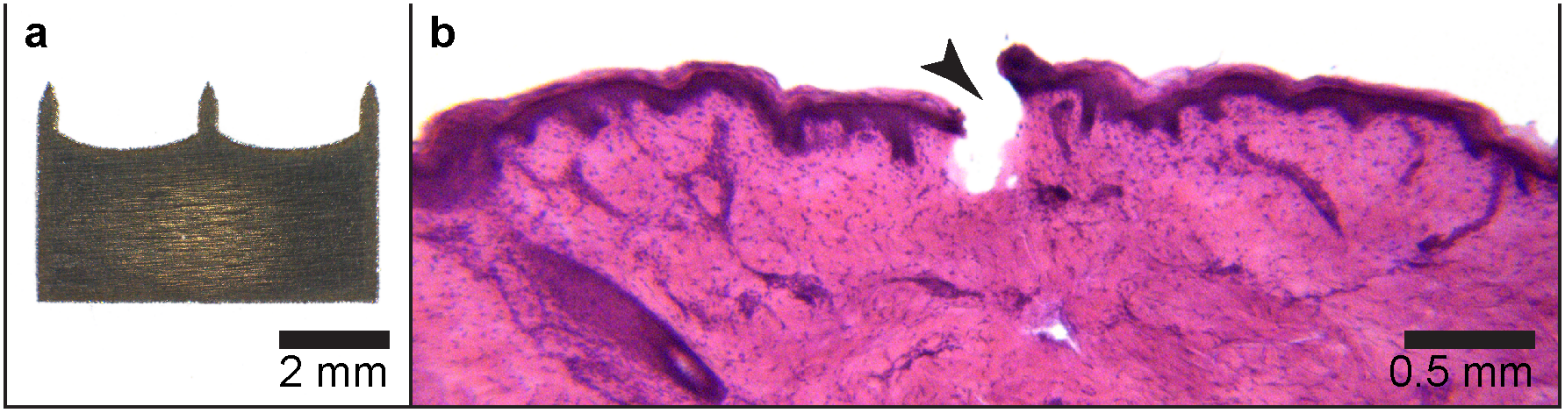
Hematoxylin and eosin image of microneedle hole into excised human skin. The image of microneedle (MN) plate used (with three 700 µm length x 250 µm with MN) is shown (a). Representative image of a 30 µm thick Hematoxylin and eosin (H&E) stained crysection of a microneeedle (MN) penetration site in human skin is shown (b). In this image the microneedle fissure (arrowhead) is 432 µm, reaching the superficial dermis.

The design of the microneedles use in this study is shown in **Figure 1** **.** The microneedles penetrated 304±63 µm deep into human skin. **Figure 1** shows a haematoxylin and eosin stained section of human skin treated with the microneedles. There is a clear puncture site that extends into the superficial dermis. This barrier breach is the best case scenario for enhancing topical peptide delivery.

### 3.2. Penetration and distribution of microneedle assisted peptides in excised human skin

#### 3.2.1 Melanostatin

**Figure 2:**
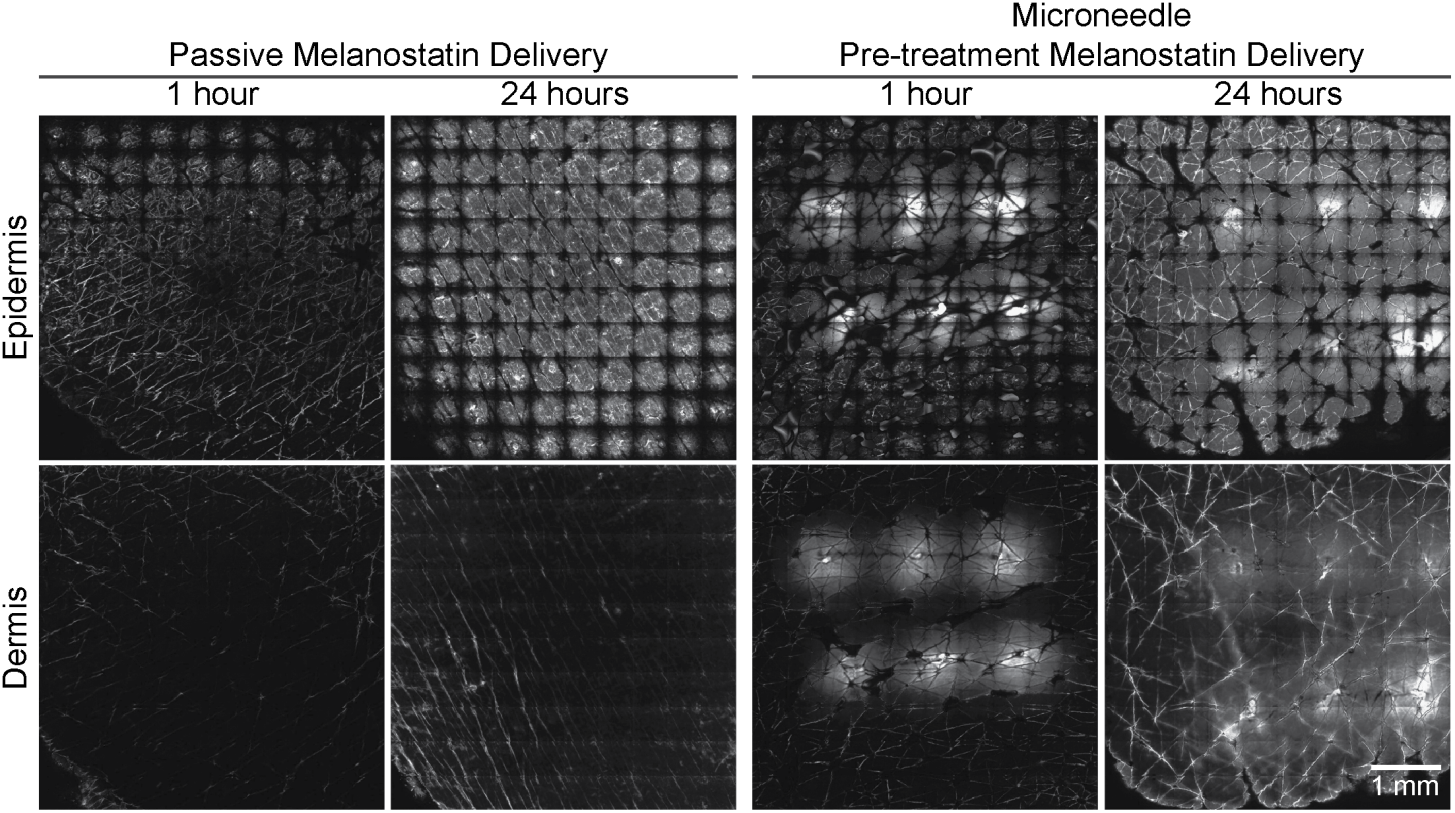
LSCM images of melanostatin delivery into excised human skin. Representative melanostatin (MEL) treated confocal laser scanning microscopy (CLSM) images of viable epidermis and dermis at 1 and 24 hour(s) are shown without and with microneedle (MN) delivery enhancement. Mosaic images of the viable epidermis and dermis (top row and bottom row, respectively) are shown at at 1 and 24 hour(s) after melanstatin delivery with microneedle enhancement. Each mosiac is 5×5 mm^2^.

**Figure 3:**
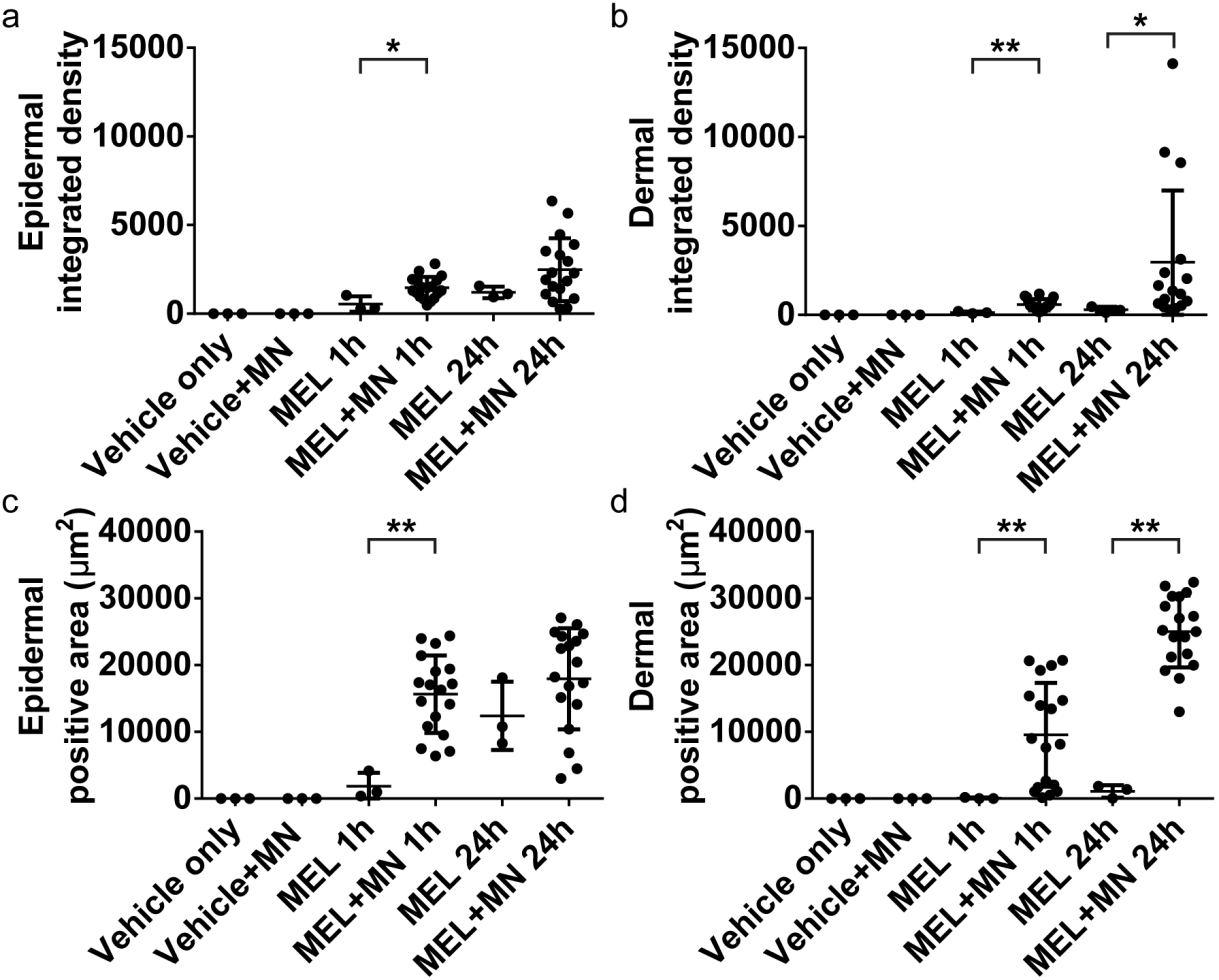
Integrated density and positive area data from melanostatin treated skin. Melanostatin (MEL) delivery characteristics are shown from epidermal (a and c) and dermal (d and d) mosiacs. Both the integrated density (a and b) and positive area (c and d) are shown for each microneedle site (MN). Data are shown for both 1 and 24 hours peptide exposure (1 h and 24 h, respecitvely). * indicates p<0.05. ** indicates p<0.01.

Melanostatin penetration into the viable epidermis and dermis with lateral diffusion can be seen in **Figure 2** **.** The fine lines seen in these images are furrows containing the fluorescently labelled peptide. Each mosaic is composed of 10×10 images at 500×500 µm each. By comparing between 1 hour and 24 hours microneedle assisted delivery, the data shows some increased diffusion of melanostatin to 24 hours. There was minimal penetration of melanostatin in both viable epidermis and dermis without microneedle pre-treatment (**Figure 2**). **Figure 3** shows image analysis outcomes that at 1 hour, microneedles enhanced Melanostatin positive area by 8.5-fold in the viable epidermis (15,648±5,802 µm^2^) when compared to without microneedles (1,842±2,025 µm^2^). This difference was found to be statistically significant (*p*<0.01). At the same time-point the integrated density in the epidermis was significantly increased by 2.6-fold with microneedles (1,463±620) and without microneedles (549±431) (**Table 1**).

**Table 1:** Values of integrated density and positive area in epidermis and dermis at 1 hour and 24 hour time points.

	Integrated density				Positive area (µm^2^)			
	Epidermis		Dermis		Epidermis		Dermis	
	1 hour	24 hours	1 hour	24 hours	1 hour	24 hours	1 hour	24 hours
**Melanostatin**	548.7±431.4	1208.0±321.4	137.0±62.2	289.3±171.8	1842±2025	12395±5117	23±97	1094±938
**Melanostatin + MN**	1463±620.4*	2487.0±1770.0	578.4±316.1**	2970±4026*	15648±5802**	17948±7578	9560±7783**	25032±5390**
**Rigin**	1325.0±613.8	2172.0±492.4	716.8±504.1	374.0±228.6	9763±3064	16392±2395	1561±906	4269±4172
**Rigin + MN**	2334.0±781.0*	1264.0±907.1*	768.0±192.7	324.8±237.0	17765±4348*	16594±6218	7683±2887**	2730±2317
**Pal-KTTKS**	255.8±167.8	3070.0±4101.0	155.3±61.0	196.9±84.1	1902±1525	6578±2441	23±14	205±208
**Pal-KTTKS + MN**	656.1±272.1	13880±22614	950.7±1403.0	323.1±573.4	977±323	4219±1742	46±54	367±419

* indicates p<0.05 and ** indicates p<0.01.

Enhancements of 2.1 and 1.4 fold were seen in the positive area and integrated density at 24 hours with and without microneedles, respectively. The trend and deviation intensified with increasing depth. Microneedle enhancement showed a significant increase in the total area of penetration in the dermis (without microneedles: 23±97 µm^2^; with microneedles: 9,560±7,783 µm^2^) after 1 hour. Although highly variable, the differences were significant (*p*<0.0001). At 24 hours the comparison was similar, a 22-fold increase in area was seen in the dermis from 1,094±938 µm^2^ without microneedles to 25,032±5,390 µm^2^ with microneedles. This was accompanied with a non-significant 1.7-fold increase in dermal integrated density after 24 hours from 196±84 without microneedles to 333±180, with microneedle delivery enhancement. Taken together, these data show that microneedles can indeed enhance topical melanostatin delivery.

#### 3.2.2 Rigin

**Figure 4:**
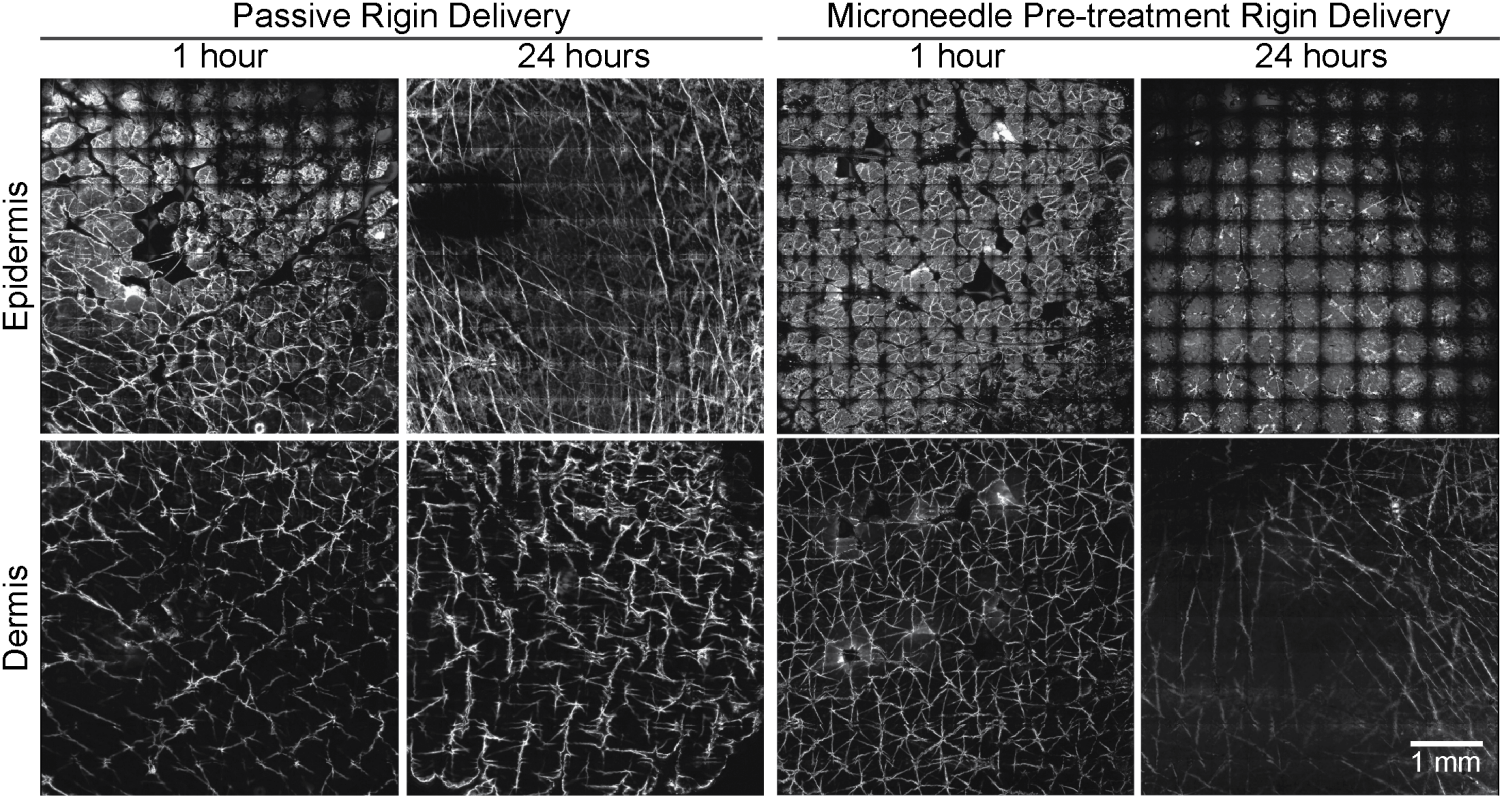
LSCM images of rigin delivery into excised human skin. Representative rigin (RIG) treated confocal laser scanning microscopy (CLSM) images of viable epidermis and dermis at 1 and 24 hour(s) are shown without and with microneedle (MN) delivery enhancement. Mosaic images of the viable epidermis and dermis (top row and bottom row, respectively) are shown at at 1 and 24 hour(s) after rigin delivery with microneedle enhancement. Each mosiac is 5×5 mm^2^.

**Figure 5:**
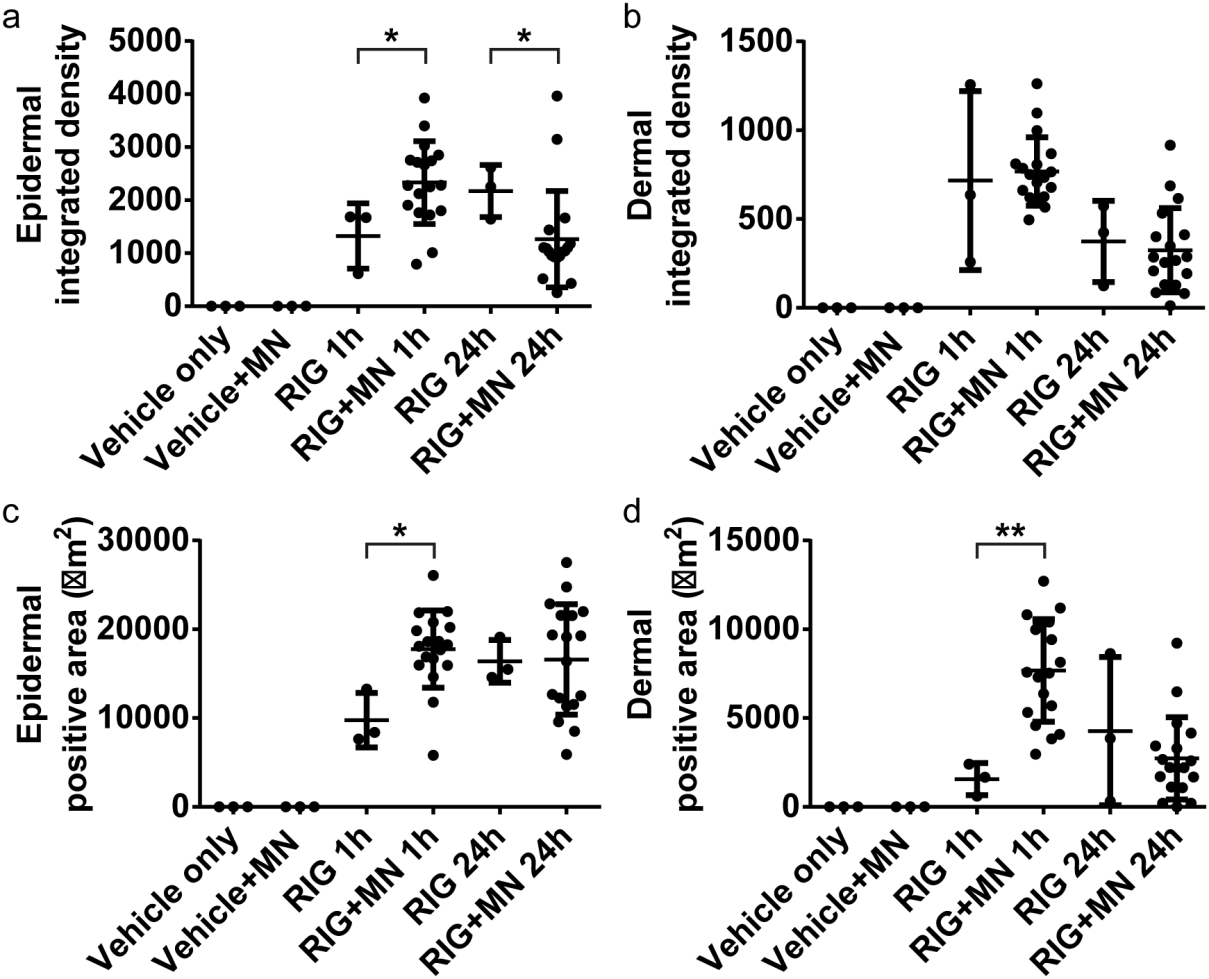
Integrated density and positive area data from rigin treated skin. Rigin (RIG) delivery characteristics are shown from epidermal (a and c) and dermal (d and d) mosiacs. Both the integrated density (a and b) and positive area (c and d) are shown for each microneedle site (MN). Data are shown for both 1 and 24 hours peptide exposure (1 h and 24 h, respecitvely). * indicates p<0.05. ** indicates p<0.01.

**Figure 4** shows the 1 and 24 hour(s) images from rigin treated skin with or without microneedle application. There was a slight increase in permeation of rigin in viable epidermis with microneedles at 1 hour compared to that without microneedles. After 24 hours post treatment, there were not substantial differences in rigin permeation at the viable epidermal level with respect to microneedle treatment. The dermal permeation was similar between microneedles and without microneedles at 1 and hours post administration (**Figure 4**). The quantification results (**Figure 5**) show Rigin with microneedles resulted a 1.8-fold increase in the positive area (Rigin 1 hour with microneedles: 17,765±4,348 µm^2^; and without microneedles: 9,763±3,064 µm^2^) and 1.7-fold increase in the integrated density in the viable epidermis at 1 hour (*p*<0.05). At 24 hours the Rigin positive areas in the viable epidermis were nearly identical with or without microneedles at 16,594±6,218 and 16,392±2,395 µm^2^, respectively (**Table 1**). The dermal results were similar in comparison with a ∼6-fold drop in Rigin positive area compared to that in the viable epidermis. These data show that microneedle penetration enhancement was effective at 1 hour post treatment, but this effect was not observed at the 24 hour time point.

#### 3.2.3 Pal-KTTKS

**Figure 6:**
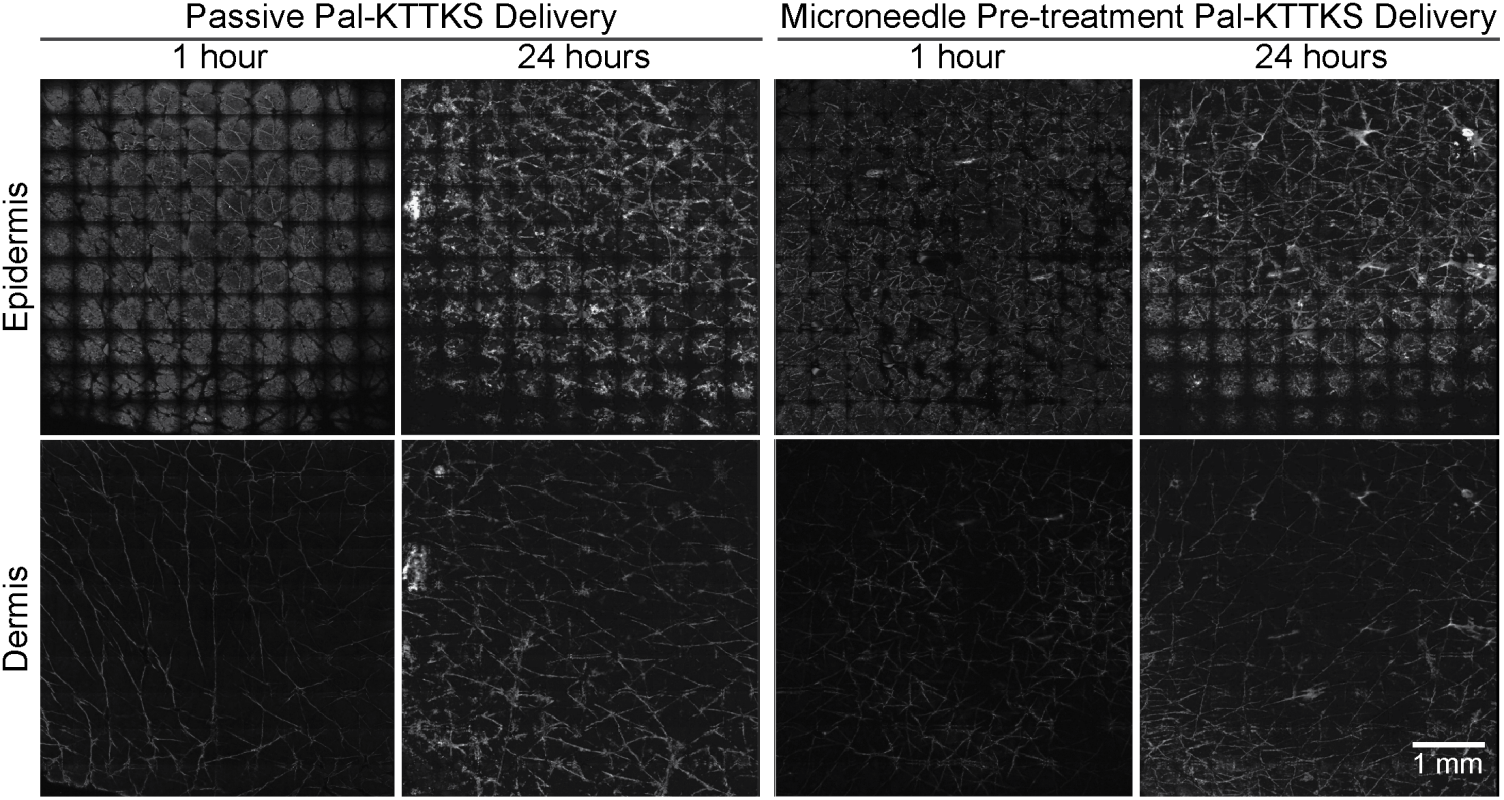
LSCM images of Pal-KTTKS delivery into excised human skin. Representative Pal-KTTKS treated confocal laser scanning microscopy (CLSM) images of viable epidermis and dermis at 1 and 24 hour(s) are shown without and with microneedle (MN) delivery enhancement. Mosaic images of the viable epidermis and dermis (top row and bottom row, respectively) are shown at at 1 and 24 hour(s) after Pal-KTTKS delivery with microneedle enhancement. Each mosiac is 5×5 mm^2^.

Following the microneedle enhanced delivery with Pal-KTTKS, the images (**Figure 6**) showed no obvious change in integrated density or penetration area in the viable epidermis at either 1 or 24 hours with or without microneedles. The outline of the microneedle penetration sites was visible for the most part, but the signal appeared limited to the immediate area.

**Figure 7:**
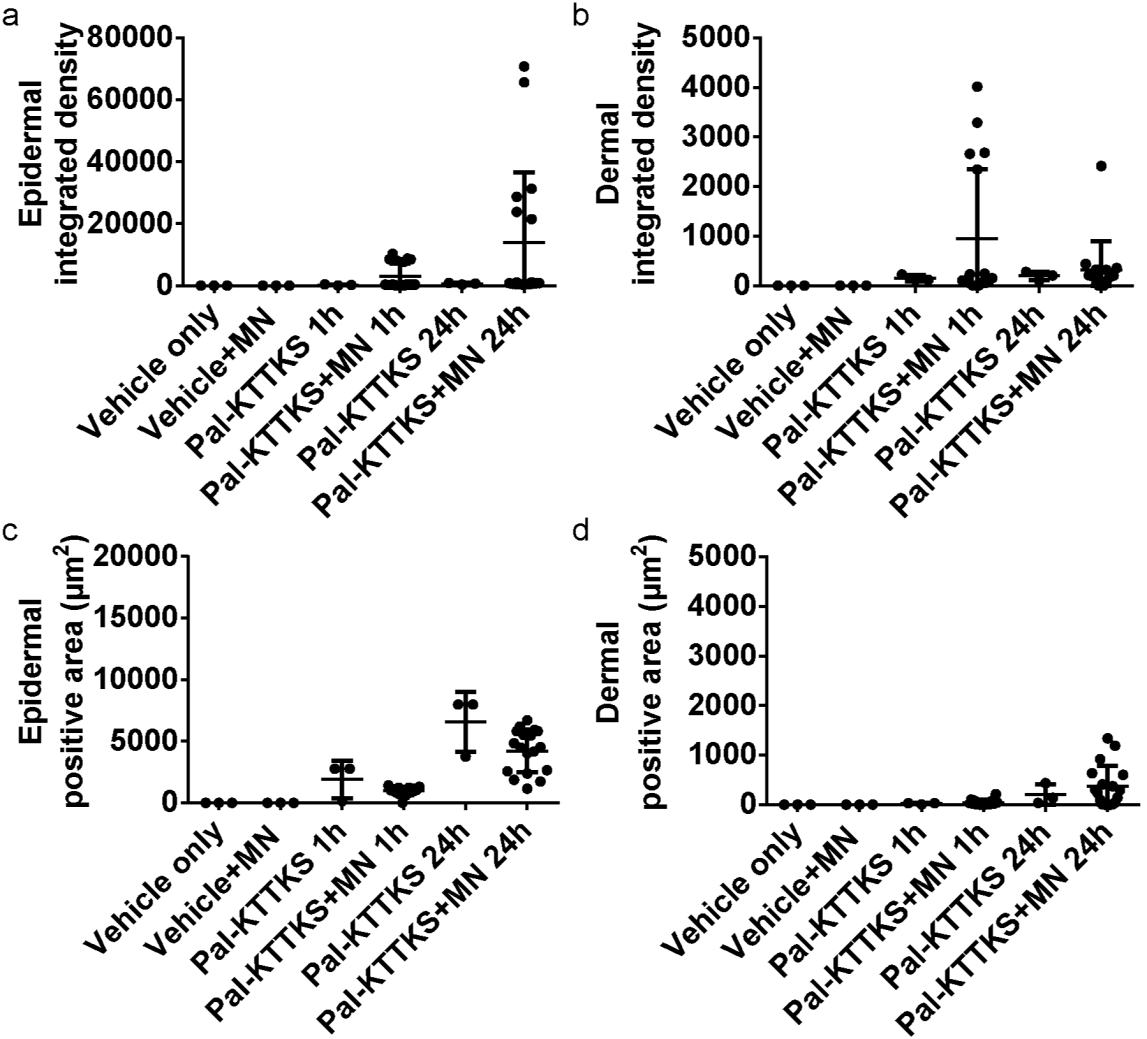
Integrated density and positive area data from Pal-KTTKS treated skin. Pal-KTTKS delivery characteristics are shown from epidermal (a and c) and dermal (d and d) mosiacs. Both the integrated density (a and b) and positive area (c and d) are shown for each microneedle site (MN). Data are shown for both 1 and 24 hours peptide exposure (1 h and 24 h, respecitvely).

Image analysis revealed no significant changes in the integrated density or positive area after 1 hour. However, after 24 hours treatment we observed a 4.5 fold increase in integrated density while the Pal-KTTKS positive area remained the same (**Figure 7**). The increase in integrated density was only observed in a subset of microneedle sites (6/18) and was therefore associated with a large standard deviation. At first we suspected the microneedle application was to blame, but this phenomena was only observed with Pal-KTTKS and not melanostatin or rigin. Additionally, the positive area measurements did not show the same trend. Therefore, we hypothesize that this observation may be due to the negligible penetration profile combined with the dynamic nature of skin pore morphology. These increased mean integrated density values were associated with large standard deviations that minimise the relevance of this perceived penetration enhancement (e.g. dermal integrated density increased from 196.9±84.1 to 323.1±573.4 with microneedle pre-treatment at 24 hours) (**Table 1**). Overall, microneedle pre-treatment did not appear to significantly and reproducibly enhance the delivery of Pal-KTTKS.

## Discussion

There is a balance between microneedles sufficiently long enough to penetrate through the skin barrier for enhanced drug delivery but small enough to cause minimal skin injury and pain. Our study showed the penetration of MN alone into epidermis. Developing clinically feasible microneedle transdermal delivery of peptides is complex. One reason for this is that after the microneedle physically enters the skin, it is almost certain that peptides can get below stratum corneum. However, the diffusion of individual peptides need to be observed so that the potential for clinical/cosmeceutical benefits can be predicted. Positive outcomes from these experiments could result in new devices as skin pre-treatment tools or skin microinjections.

The field of microneedle enhanced protein delivery is largely focused on insulin and vaccine delivery. For a review see Kim *et al.* [12]. Insulin is a protein composed of 51 amino acids that has a molecular weight of 5808 Da. Therefore, comparing microneedle enhanced insulin delivery to even the largest peptide in this study, Pal-KTTKS-fluorescein conjugate at 1191.06 Da, is not relevant. However, there are many reports of enhanced transdermal peptide delivery using approaches other than microneedles (for review see Benson and Namjoshi [13]) and a handful of reports with microneedle enhanced peptide delivery.

A recent report by Sachdeva *et al.* investigated the use of iontophoresis with and without microneedles to enhance the topical delivery of leuprolide [14]. This 9 amino acid containing peptide has a molecular weight of 1209.40 Da, which is similar in mass to our melanostatin (803.92 Da), rigin (959.04 Da) and Pal-KTTKS (1191.06 Da) -fluorescein conjugates. Both peptides require penetration enhancement to cross the skin barrier. Sachdeva *et al.* found that leuprolide penetrated to blood levels of 0.36±0.22 ng/ml after 6 hours without enhancement. The authors subsequently found that microneedle application improved delivery by only 2.7 fold. Similarly, we found that at 1 hour post treatment we observed a 4.2 (melanostatin), 1.1 (rigin) and 6.1 (Pal-KTTKS) fold increase in dermal signal within the microneedle pre-treated groups.

These similarities in fold increase were quite comparable considering differences in the peptide sequences, models, microneeldes and detection approaches. This low level improvement supports the hypothesis that enhancing the transdermal delivery of some peptides requires more than just microneedle holes in the skin.

Sachdeva *et al.* also described iontophoresis as a more effective means to enhance leuprolide delivery across rat skin (9.6 fold enhancement over passive treatment) than microneedles. This suggests that the dissolving microneedles used in the Sachdeva *et al.* study may have been blocking the diffusion of the peptide through the relatively thin rat skin and iontophoresis helped overcome the skin barrier and/or that passive diffusion, even with perforated skin, was still negligible. The combination of the two technologies only improved delivery over iontophoresis alone by 1.02 fold. This modest improvement suggests that iontophoresis was key in moving leuprolide across the rat skin barrier. Iontophoresis may also improve Pal-KTTKS in human skin, but this has yet to be reported. In contrast, we found that microneedles were highly effective in improving transdermal melanostatin delivery, highlighting the necessity to tailor the penetration enhancing technology to the particular peptide of interest.

Desmopressin is a another 9 amino acid long peptide that has been investigated for transdermal delivery with microneedle enhancement and has a molecular weight of 1069.22 Da. Cormier *et al.* used a microneedle array made from titanium that was dry coated with desmopressin formulated in 0.2 wt % polysorbate 20. The study was carried out in hairless guinea pigs. The microneedles were 200 µm long and had a maximal width of 170 µm. Our microneedles were 500 µm longer and 80 µm wider, but we used only 6 microneedles whereas Cormier *et al.* used 642 microneedles. This means that a projected microneedle area impacting the skin for our study was 0.075 mm^2^ per group versus 3.8 mm^2^ in Cormier *et al.*

Cormier *et al.* did not report desmopressin penetration without microneedles, so we cannot easily compare penetration enhancement. However, they did measure variability in the microneedle experiments. Cormier *et al.* calculated that the microneedle array was capable of delivering 17.5±3.8 µg desmopressin in a single application. The standard deviation equals 21.7% of the mean delivered dose. We found that the integrated density of our 6 microneedle array delivery approach varied from 8.3% (melanostatin), 10.8% (rigin) to 30.9% (Pal-KTTKS) of the mean value after 1 hour in the epidermis. Deviation also increased with depth and time in our study.

There is an evident trend with increasing molecular weight and variability within our data set. There appears to be less variability in the Cormier *et al.* data compared to our Pal-KTTKS and more than we found with rigin and melanostatin. This could be due to peptide diffusion and could also be influenced by the differences in the delivery approaches. Cormier *et al.* used coated microneedles, whereas we employed a “poke and rub” approach. They had over 100 times more microneedles in their device than we had. This could have resulted in a reduced impact of imperfect microneedle penetration. Alternately, the vertical and horizontal diffusion characteristics of the individual peptides within the different skin strata could have also contributed to variability.

## Conclusions

In conclusion, we have demonstrated that microneedles can be effective way of enhancing some large and complex pharmaceutically active molecules deep into the skin. The data correlate with previous reports despite considerable technical differences between studies. We observed that the lowest molecular weight peptide was associated with the most improved topical delivery enhancement using microneedle pre-treatment. We also observed that the delivery of a larger molecular weight peptide was not improved by microneedle pre-treatment. Therefore, using microneedle penetration enhancement could be effective when delivering some therapeutic peptides, but microneedle pre-treatment is not a one size fits all solution for topical delivery.
